# Rare Case of Lower Gastrointestinal Bleeding Secondary to Miliary Tuberculosis in the United States

**DOI:** 10.7759/cureus.57177

**Published:** 2024-03-29

**Authors:** Marta B Sekh, Alexa B Jack, Danielle A Rowe, Nitori G Henderson, Michael R Zemaitis

**Affiliations:** 1 Medicine, College of Medicine, American University of Antigua, Osbourn, ATG; 2 Medicine, St. George's University School of Medicine, St. George's, GRD; 3 General Surgery, Richmond University Medical Center, New York City, USA

**Keywords:** abdominal tb, gastrointestinal tuberculosis (gitb), lower gastrointestinal bleeding secondary to tuberculosis, extrapulmonary tuberculosis (eptb), miliary tb

## Abstract

Tuberculosis (TB) remains a significant global health challenge. Miliary TB is a rare manifestation of TB that involves systemic lymphohematogenous dissemination of infection and presents diagnostic challenges due to its often asymptomatic or non-specific nature. This case report documents a rare occurrence of gastrointestinal (GI) bleeding secondary to miliary TB without pulmonary symptoms in an 81-year-old Filipino-American male living in the United States. Extensive imaging studies revealed a mass in the right colon with multiple bleeding vessels draped around it; it was not amendable to treatment with embolization and required right hemicolectomy with end ileostomy. The pathology report of the excised mass demonstrated miliary TB with necrotizing granulomas and granulomatous lymphadenopathy involving 23 lymph nodes. The patient was started on anti-tuberculosis medical management; however, the patient remained clinically unstable and expired on postoperative day 39. This case highlights the importance of the heightened clinical awareness required during times of globalization and in regions with dense immigrant populations. We aim to delineate the clinical understanding of gastrointestinal TB (GITB) and review possible indications for surgical management. We aim to help reduce diagnostic delay, therefore improving patient outcomes and limiting the spread of disease.

## Introduction

Tuberculosis (TB) is the second-leading infectious killer after COVID-19 worldwide. An estimated two billion people are asymptomatic carriers of TB infection. In 2022, 10.6 million people developed TB disease, and 1.3 million people died from it [1]. According to the latest CDC report, up to 13 million Americans are living with latent TB infection, and 8,331 new cases of TB were reported in 2022 [2]. Notably, the miliary form of TB is rare and accounts for only 1%-2% of all TB diseases [3].

Tuberculosis is an infectious disease caused by *Mycobacterium tuberculosis* bacilli that most commonly affects the lungs, and, at times, the hematogenous spread of the infection may lead to the development of extrapulmonary TB (EPTB). Extrapulmonary TB can occur in isolation or alongside pulmonary TB. In rare cases, severe systemic lymphohematogenous dissemination may occur when pulmonary or extrapulmonary foci containing *M. tuberculosis* bacilli rupture and embolize into the vessels of various organs, leading to a morphologically distinctive form of TB known as miliary TB. Less commonly, miliary TB can also result from the simultaneous reactivation of latent TB in various organs. The term 'miliary’ describes the pathologic and/or radiologic appearance of small, firm nodules that resemble millet seeds, which is pathognomonic for miliary TB. Clinically, miliary TB is often asymptomatic. When symptoms are present, they are mostly non-specific (cough, dyspnea, constitutional symptoms, lymphadenopathy, hepatosplenomegaly, etc.) or atypical, which delays the diagnosis. Miliary TB is fatal if not diagnosed and treated on time; thus, a definitive diagnosis of miliary TB is often done post-mortem [3, 4]. Despite the vast systemic involvement of miliary TB, the prevalence of abdominal TB is only 5%, and gastrointestinal TB (GITB) is as low as 1-3% [4,5]. Gastrointestinal TB can affect any part of the tract, from mouth to anus, and can mimic any number of gastrointestinal (GI) pathologies. The clinical presentation of GITB can range from fever, abdominal pain/distention, ascites, and diarrhea to more serious complications like GI bleeding, bowel obstruction, and perforation. Most patients respond well to standard anti-tuberculosis therapy; however, diagnostic delays and frequent misdiagnosis can necessitate surgical intervention secondary to complications [6].

This case report illustrates a rare presentation of lower GI bleeding secondary to miliary TB in the United States. The significance of this case report lies not only in its two-fold rarity but also underscores the increasing need for awareness required during globalization and increasing immigration from endemic areas. This treatable and preventable disease poses challenges in non-endemic areas where a lack of clinical suspicion may lead to delayed diagnosis and may necessitate urgent surgical intervention.

## Case presentation

An 81-year-old Filipino-American male with a medical history of rheumatoid arthritis (on methotrexate, prednisone, and non-steroidal anti-inflammatory drugs), benign prostatic hyperplasia, peripheral artery disease, and hyperlipidemia presented to the emergency department (ED) with a chief complaint of two episodes of syncope. The patient’s daughter-in-law witnessed the first episode after lunchtime, which lasted for one minute. The second episode occurred around dinnertime with associated emesis and pallor, which prompted the activation of emergency medical services (EMS). On the way to the ED, the patient had two more episodes of non-bloody emesis of GI contents. The patient also had an episode of bright red bleeding per rectum (BRBPR) at home and another one (around 200 cc) in the ED. Reportedly, the patient had a similar episode of BRBPR one month ago, for which a colonoscopy was done at a different hospital. The bleeding source was localized and cauterized at that time. The specifics of the colonoscopy were unknown. 

In the ED, the patient was afebrile (97.5°F) with a heart rate of 102 beats per minute, blood pressure of 97/62 mmHg, respiratory rate of 18 breaths per minute, and oxygen saturation (SpO2) of 94% on room air. On the physical exam, the patient was lethargic and in moderate distress, but he responded to verbal stimuli. There was conjunctival and body pallor, normal bowel sounds, no tenderness on abdominal palpation, and no edema. Laboratory findings (Table 1) were significant for low hemoglobin, hematocrit, and platelets, mild hyponatremia, hypercalcemia, elevated blood urea nitrogen, creatinine, and glucose levels. Initial chest X-rays (CXR) and contrast-enhanced computed tomography (CECT) of the abdomen and pelvis were unremarkable. In the ED, this patient received intravenous fluids, two units of packed red blood cells (pRBCs), and intravenous pantoprazole. Admission to the medical intensive care unit was required for the management of acute blood loss, anemia secondary to lower GI bleeding, acute kidney injury secondary to blood loss, and hypercalcemia.

**Table 1:** Laboratory values procured in the emergency department

Investigation	Patient’s laboratory values	Reference ranges
Hemoglobin	7.8 g/dL	13.5-17.5 g/dL
Hematocrit	24.7%	40%-51%
Platelet	112,000 k/ul	150,000-400,000 k/ul
Serum sodium	134 mmol/L	136-145 mmol/L
Serum calcium	11.7 mg/dL	8.3-10.6 mg/dL
Serum blood urea nitrogen	69 mg/dL	7-18 mg/dL
Serum creatinine	1.7 mg/dL	0.7-1.30 mg/dL
Serum glucose	136 mg/dL	74-106 mg/dL

**Figure 1:**
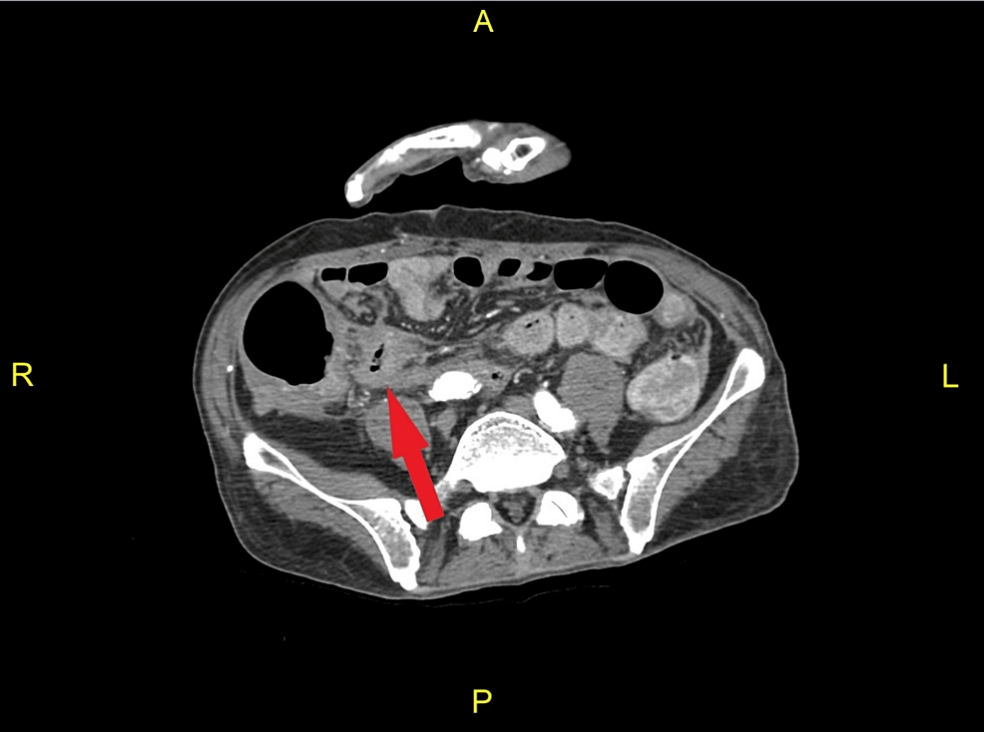
A CT angiogram of the abdomen and pelvis (axial section) Apparent long-segment mucosal thickening of the terminal ileum (red arrow) suggests chronic inflammation, possibly Crohn’s disease. There is no identified source of active bleeding. There is abundant fecal material throughout the rectum and sigmoid colon.

**Figure 2:**
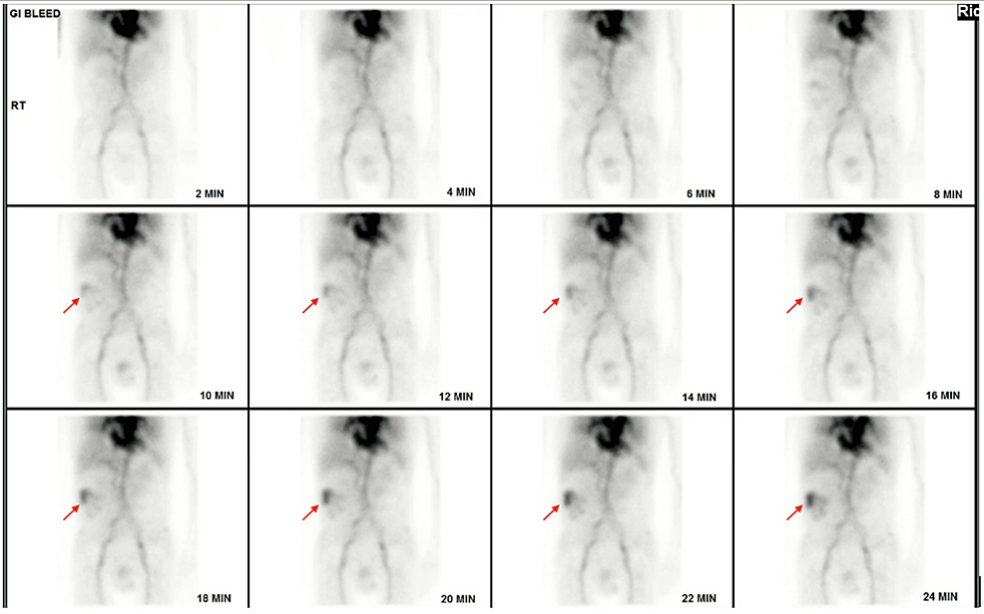
Nuclear medicine GI bleed scan The accumulation of abnormal activity in the right flank (red arrow) over the course of the study suggests an acute hemorrhage into the ascending colon.

A CT angiography of the abdomen and pelvis with and without contrast (Figure 1) was performed, but it did not identify a source of active intestinal bleeding. However, apparent long-segment mural thickening of the terminal ileum was reported, suggesting chronic inflammation possibly due to Crohn’s disease. No obstruction was identified; only abundant fecal material throughout the rectum and sigmoid colon was noted. A nuclear medicine scan (Figure 2) was subsequently performed, which demonstrated focal uptake in the region of the cecum consistent with the impression of an acute hemorrhage in the ascending colon, which was localized to the area, or, in other words, a bleeding mass detected. The patient was then taken to the interventional radiology (IR) suite for mesenteric angiography, with the goal of embolization. Their findings confirmed a mass in the right colon with multiple feeding vessels draped around it. However, they did not identify any actively bleeding vessels that qualified for selective embolization. The IR team suggested a colonoscopy and a surgical consultation for further advice. The patient’s prior colonoscopy report from one month ago was reviewed, which reported a near circumferential cecal mass with findings concerning malignancy.

**Figure 3:**
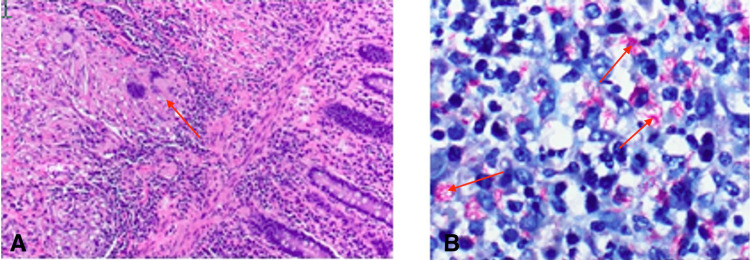
Histopathology of the right colon specimen (A) hematoxylin and eosin stain (20x) reveals caseating granuloma (red arrow); (B) acid-fast stain positive for bacilli (red arrows)

The patient was hemodynamically unstable on pressors with continued transfusion requirements and passing blood clots per rectum; therefore, surgical intervention was required. Once stabilized, the initial plan for laparoscopic right hemicolectomy was abandoned upon entry into the abdomen due to presumed diffuse carcinomatosis affecting the small and large bowels, liver, and abdominal wall. The peritoneum was fibrotic and challenging to handle. Consequently, the procedure was converted to open surgery. The subsequent intraoperative course was uneventful; the segment of bowel from the terminal ileum to the proximal one-third of the transverse colon was resected (terminal ileum, cecum, appendix, ascending colon, proximal one-third of the transverse colon) and sent for pathology. An end ileostomy was created in favor of primary anastomosis due to the extent of the disease. On postoperative day (POD) seven, the pathology report of the excised bowel indicated miliary TB involving small and large intestines with mucosal ulceration and numerous submucosal, serosal, and omental necrotizing granulomas (Figure 3a) and acid-fast positive bacilli (Figure 3b). Granulomatous lymphadenopathy was present in 23 lymph nodes. The patient was placed on airborne precautions, and the infectious diseases department was consulted for further management. The added medical regimen included rifampin, isoniazid, pyrazinamide, ethambutol (‘RIPE’ therapy), and pyridoxine.

By POD 12, the patient was successfully extubated, the ileostomy was functioning well, and two abdominal Jackson-Pratt drains were removed. However, the course of recovery was complicated by incisional wound dehiscence, intraabdominal abscess, which was treated with abdominal washout, and bleeding from operative sites despite pharmaceutical interventions. The patient continued to have episodes of hypotension, with systolic pressure ranging in the 50s and tachycardia requiring pressors and blood products. The family agreed to palliative care without aggressive measures or activation of any transfusion protocols, and the patient was removed from the ventilator to allow for natural death. The patient died after cardiopulmonary arrest on POD 39. 

## Discussion

Risk factors

The risk factors for developing TB include diabetes, renal disease, a weakened immune system (HIV or AIDS), underlying malignancy, tobacco use, and malnourishment. However, a significant number of patients with miliary TB do not have any underlying risk factors, and only 15%-25% of abdominal cases of TB have pulmonary involvement [1,4,6]. As in our case, the patient denied a history of smoking or alcohol use but was on immunosuppressive medication for rheumatoid arthritis. His heritage may have possibly been a significant risk factor, as the Philippines is the fourth largest contributor to global TB cases [7]. However, he denied travel within the last 21 days of his ED presentation. Due to the lack of underlying risk factors and pulmonary symptoms, miliary TB is easy to misdiagnose in areas with low TB prevalence, such as the United States.

Pathophysiology

Although miliary TB can have widespread systemic involvement, abdominal TB is uncommon. Abdominal TB can involve lymph nodes, peritoneum, GI tract (GIT), and visceral organs. *Mycobacterium tuberculosis* bacilli can infect the abdomen through multiple modes, including ingestion of contaminated milk or sputum, which allows the bacilli to infect the mucosal layer of the GIT and form epithelioid tubercles in the lymphoid tissue of the submucosa. Over two to four weeks, the caseating necrosis of the tubercles leads to mucosal ulceration and promotes spreading into the deeper layers as well as the lymphoid tissue and the peritoneum. In some rare cases, these bacilli can get into the portal circulation or hepatic artery and spread into the solid organs of the GIT [8]. Other modes of abdominal TB infection include hematogenous spread from tubercular foci elsewhere in the body, direct spread from the infected adjacent foci, and lymphatic spread from infected lymph nodes through the lymphatic channels [6].

The most common site of GITB involvement is the ileocecum (64% of all cases), followed by the jejunum and colon, while the esophagus, stomach, and duodenum are rarely involved. In accordance with these data, our patient’s gross pathology examination revealed a large lesion (8.5 cm in width, 4 cm in length) involving the ileocecal valve, distal terminal, and proximal ascending colon and cecum. This lesion consisted of gray tissue involving the entire thickness of the wall, with notable peritoneal and lymph node involvement. The serosal surface of the terminal ileum and right colon had diffused small, whitish granulomas ranging in size from 0.1 cm to 0.5 cm. The ileocecal region may be a preferential site for TB due to its high physiologic stasis, high electrolyte, and fluid reabsorption, minimal digestive activity, and abundance of lymphoid tissue. The frequency of involvement decreases as you move proximally and distally away from the ileocecal region [6, 9]. Therefore, clinical symptoms of GITB vary depending on the site of involvement and the extent of the disease.

Clinical presentation

The clinical presentation of GITB can be acute, chronic, or acute-on-chronic, often lasting from weeks to months. Individuals can present with fever, abdominal pain, diarrhea, constipation, altered bowel habits, weight loss, anorexia, and malaise. The pain can be colicky due to the narrowing of the lumen or dull and constant when the disease affects mesenteric lymph nodes [9]. When complications ensue, the patient can present with blood in the stool, strictures or fistula formation, intestinal obstruction, and/or perforation [6,10,11]. On ED admission, our patient endorsed nausea, vomiting, and hematochezia and denied abdominal pain, diarrhea, or altered bowel habits. He also denied fever, coughing, shortness of breath, and chest pain. In the absence of pulmonary symptoms, the clinical suspicion of inflammatory bowel disease (IBD), such as Crohn’s disease (CD), can be greater than that of GITB [12]. To improve clinical recognition of miliary TB, a retrospective study analyzed autopsy cases of clinically unrecognized miliary TB to identify the clinical diagnosis that most frequently masks the underlying TB. They found that the most common diagnoses that mask pulmonary miliary TB are chronic obstructive pulmonary disease (COPD) and pulmonary thromboembolism, while generalized miliary TB is most often misdiagnosed as adult respiratory distress syndrome, sepsis, GI bleeding, and meningoencephalitis. These data were from specialized pulmonary hospitals in Serbia, which show that miliary TB poses diagnostic challenges even to the most skilled clinicians [4].

Diagnostics

When diagnosing GITB with concomitant pulmonary involvement, a sputum culture and a CXR are the simplest investigations. Although a sputum culture positive for acid-fast bacilli (AFB) and a CXR with evidence of tuberculosis support the diagnosis of GITB, it does not rule it out. The definitive diagnosis of GITB can be made by detecting *M. tuberculosis* in the peritoneal fluid in the presence of ascites, a biopsy specimen from the involved site, or via mycobacterial culture and/or nucleic acid amplification test (NAAT) [9].

Radiological studies are particularly useful in the diagnosis of miliary TB. A CXR may reveal a miliary pattern, a collection of tiny discrete pulmonary opacities that are uniform in size, widespread in distribution, and 2 mm or less in diameter. In some patients with miliary TB, CXR may be normal initially, but this may change over the course of the disease. However, in 50% of patients with miliary TB, the classic miliary pattern may not be seen at all [3]. When CXR is normal, as in the case of our patient, the clinical suspicion of TB dramatically decreases. High-resolution CT scans improved the antemortem diagnosis of miliary TB and may reveal a classic miliary pattern even when CXR appears normal. It may show interlobular septal thickening, intralobular fine network, and/or centrilobular nodules and branching linear structures. Despite this, one must remain critical when diagnosing miliary nodules on radiological imaging alone, as they may also occur in conditions such as sarcoidosis, silicosis, extrathoracic malignancy, and histoplasmosis [13]. Contrast-enhanced CT and magnetic resonance imaging (MRI) are useful for detecting miliary TB lesions at extrapulmonary sites [3]. In the case of GITB, depending on the progression of the disease, CT may show wall thickening up to 3 cm in the cecum and terminal ileum with mesenteric lymphadenopathy and features of intestinal obstruction and/or perforation [6]. Interventional radiology procedures such as fine needle aspiration for cytology and an image-guided biopsy can confirm the diagnosis. Exploratory laparotomy may also be performed for intraoperative specimen collection [9]. As in our case, the patient was hemodynamically unstable with continual BRBPR with clots per rectum, necessitating an exploratory laparotomy, which allowed for gross and pathohistological analysis of the excised mass. The biopsy was positive for AFB and showed caseating granuloma, confirming a miliary TB diagnosis. To further confirm the diagnosis, a sputum culture was done postoperatively, which came back positive for *M. tuberculosis* bacilli.

Treatment

Gastrointestinal TB is very responsive to medication management alone when diagnosed and treated early. All cases of GITB should receive at least six months of anti-tuberculosis therapy, which includes variable regiments of RIPE therapy. Surgical intervention can also be required when complications secondary to GITB arise, such as massive GI bleeding, non-resolving intestinal obstruction, perforation, abscess, or fistula formation [6]. Noteworthy, obstructions can arise or worsen during medical management as the tissue heals by cicatrization, causing lumen narrowing and strictures [14].

When GITB is non-amendable to medical management alone, as in our case, three types of surgeries can be performed for the treatment of GITB. The first surgical option is bypassing the involved segments of the bowel by enteroenterostomy or ileotransverse colostomy. However, this approach may lead to blind loop syndrome, and strictures in the remaining segments may produce fistulas or obstructions. Thus, these surgeries are not routinely performed. Another type of surgery that attempts to eradicate the disease locally is right hemicolectomy or wide bowel resection. Nonetheless, because lesions in GITB are often widely placed, resection may not be suitable in all cases. Postoperative complications are also associated with this approach and may require re-operation for recurrent obstruction. Additionally, due to the extensive nature of this resection, patients who are malnourished, hypoproteinemic, anemic, and/or toxemic may not be able to tolerate this procedure [6, 9]. With that being said, limited resection of the ileocecal region with end-to-end anastomosis can be performed, as it requires only a small incision and less mobilization of the colon. In a segment of the intestine with multiple lesions, ileoplasty may be indicated as well [15]. The last surgery type, and the most recommended due to its conservative nature, is strictureplasty. To avoid major resection, stricturoplasty is performed for patients with multiple short-segmented strictures that cause more than 50% luminal compromise [6]. When possible, this approach may be superior because it does not sacrifice healthy bowels and has a low risk of short-bowel or blind loop syndromes [14].

## Conclusions

This case report highlights the rarity of lower GI bleeding secondary to miliary TB in non-endemic areas, such as the United States, while underscoring the importance of swift diagnosis and treatment to avoid adverse patient outcomes. Although rare, surgical interventions are sometimes required to assist in the diagnosis and treatment of miliary TB. Increased awareness of this disease among healthcare professionals working with high-risk populations and miliary TB should be considered whenever presented with a similar case as ours.
